# Locating Chart Choice Based on the Decision-Making Approach

**DOI:** 10.3390/ma15103557

**Published:** 2022-05-16

**Authors:** Vitalii Ivanov, Frantisek Botko, Vitalii Kolos, Ivan Pavlenko, Michal Hatala, Katarzyna Antosz, Justyna Trojanowska

**Affiliations:** 1 Department of Manufacturing Engineering, Machines and Tools, Faculty of Technical Systems and Energy Efficient Technologies, Sumy State University, 2, Rymskogo-Korsakova St., 40007 Sumy, Ukraine; v.kolos@tmvi.sumdu.edu.ua; 2 Department of Automobile and Manufacturing Technologies, Faculty of Manufacturing Technologies with a Seat in Presov, Technical University of Kosice, 1, Bayerova St., 080 01 Presov, Slovakia; frantisek.botko@tuke.sk (F.B.); michal.hatala@tuke.sk (M.H.); 3 Department of Computational Mechanics Named after V. Martsynkovskyy, Faculty of Technical Systems and Energy Efficient Technologies, Sumy State University, 2, Rymskogo-Korsakova St., 40007 Sumy, Ukraine; i.pavlenko@omdm.sumdu.edu.ua; 4 Department of Manufacturing Processes and Production Engineering, Faculty of Mechanical Engineering and Aeronautics, Rzeszow University of Technology, 12, Powstancow Warszawy Sq., 35-959 Rzeszow, Poland; katarzyna.antosz@prz.edu.pl; 5 Department of Production Engineering, Faculty of Mechanical Engineering, Poznan University of Technology, 3, Piotrowo St., 60-965 Poznan, Poland; justyna.trojanowska@put.poznan.pl

**Keywords:** sustainable manufacturing, flexible fixture, decision making, product innovation, CAFD, production planning, locating chart

## Abstract

Modern manufacturing engineering requires quick and reasonable solutions during the production planning stage, ensuring production efficiency and cost reduction. This research aims to create a scientific approach to the rational choice of a locating chart for complexly shaped parts. It is an important stage during the manufacturing technology and fixture design process. The systematization of the designed and technological features of complexly shaped parts and the definition of the features that impact a locating chart create the fundamentals for justification. A scientific approach has been developed using the complex combination of the part’s features and a decision-making approach using the example of bracket-type parts. The matrix of design and technological features of parts was developed including steel AISI 3135 and cast iron DIN 1691. The classification of locating charts for bracket-type parts was defined. A mathematical model of the rational choice of the locating chart according to the structural code of the workpiece was verified in case studies from the practice. As a result, a decision-making approach was applied to the rational choice of the locating chart for any bracket-type part. The proposed solutions improve the production planning stage for machine building, automotive, and other industries.

## 1. Introduction

Up-to-date production is characterized by increasing requirements for the quality and accuracy of machine parts manufacturing. One of the key directions is the precision machining of complexly shaped parts from different materials according to the quality requirements. The importance of fixture design is an urgent issue during manufacturing, which affects its productivity and flexibility.

According to current trends in creating highly automated, flexible manufacturing, there is a need for new methodological approaches to ensure quality parameters and reduce the cost of engineering products [1]. Fixtures are an integral part of the closed-loop technological system, that is, “machine tool–fixture–cutting tool–workpiece”, and are designed for accurately locating and reliably clamping workpieces during the machining of metal-cutting machine tools. Fixtures significantly affect the production of competitive engineering products, as evidenced by the following data:: fixtures make up 70–80% of the total quantity of tooling [2]; the share of fixtures is 10–20% of the total cost of the production system [3]; 80–90% of the time spent on production planning corresponds to the design and manufacture of fixtures [2]; 40% of defective parts after machining are caused by the imperfection of the fixtures [4]; 70% of new fixture layouts are modifications of existing ones [5].

The material of the workpiece influences the machining modes. Simultaneously, machining conditions impact the proper selection of the locating chart. Therefore, locating chart selection is essential to the production planning stage during manufacturing process design. Therefore, locating chart choice should also include the impact of materials for bracket-type parts. Steel and cast iron are used to make such parts in the automotive industry [6].

This research aims towards the creation a scientific approach to the rational choice of locating chart for complexly shaped parts based on a complex combination of the part’s design and technological features and a decision-making approach using the example of bracket-type parts. To achieve this goal, it is necessary to solve the following tasks. First, the matrix of the design and technological features of the parts should be proposed. Second, the classification of locating charts for bracket-type parts should be defined. Additionally, a mathematical model of the rational choice of the locating chart according to the structural code of the workpiece should be developed. This model should be verified using case studies from practice. Finally, a decision-making approach should be applied to the rational choice of the locating chart for any bracket-type part.

Process planning is recognized as the critical problem between design and manufacturing in a computer-integrated manufacturing system. Recently, many types of computer-aided process planning systems using expert system techniques have been developed [7]. Numerous research studies have been performed on computer-aided fixture design for prismatic parts. In the research study [8], the fixture modeling module was developed to assign the appropriate locating and clamping data for the prismatic part using information from the process/setup plan and the part geometrical database. In the study [9], the integrated method was developed for locating prismatic parts using the 3–2–1 locating principle. Three locating surfaces were identified for each setup to avoid the stack-up of errors. The proposed method has also considered the geometrical rules and the principles of determining the tool access directions for the feature possessing multitool access directions. Locating is an important step during fixture design. Therefore, the methodological fundamentals and mathematical apparatus should be adequately developed to ensure the design of efficient fixture configurations [10]. The research study [11] emphasizes a computer-aided fixture design system that allows for the selection, modification, and design of fixture layouts for modular fixtures. The fixture classifier for manufacturing, workpiece, locating, and clamping characteristics were identified and described. Particularly, the system allows for 3–2–1 and 4–1–1 locating principles typically used for prismatic parts and shafts. The paper [12] deals with the problem of setup and fixture planning for machining box-shaped parts on horizontal machining centers. In the proposed system, four locating charts and nine implementation methods were used for fixture design. The research study [13] presents an approach to developing intelligent design support environments for mechanical transmission systems to reduce the time between gear-tooth creation, detailed design, and final production. It provides performance evaluation, process optimization, and manufacturability analysis, and it provides reasoning and decision-making capabilities.

Implementing a decision-making approach is a reasonable solution for finding a suitable option for the research tasks. Decision-making methods have proven to be an effective support for engineering design. The multi-objective genetic algorithms are well-suited to a service-oriented architecture implementation. The research study [14] presents a grid-enabled framework for multi-objective optimization using genetic algorithms to aid decision-making in engineering design. The authors developed and systematized the methods and models for decision-making in systems engineering for developing distributed organizational information and control systems [15]. To minimize the production costs and maximize the conformity rate associated with the assembly scenario, the paper [16] presents the original method for selecting assembly techniques and allocating component geometrical tolerances for solving a multi-objective optimization problem. The study [17] presents engineering decision-making on pipe-stress analysis through the implementation of knowledge-based systems. The paper [18] describes decision-making methods for engineering design and proposes the efficiency of their use in the automotive industry.

Virtual reality has proven to be a popular technology for engineering design and maintenance, providing novel ways for visualization and interaction. The paper [19] discusses a set of application areas for VR in the industry and describes implementing a lightweight VR system for industrial engineering applications.

It should be noted that fixture design is a time-consuming process that requires a variety of information considering the capabilities of technological equipment [20], tooling [21], quality indicators [22], working conditions [23], material properties [24], etc.

## 2. Research Methodology

### 2.1. Design Requirements

Bracket-type parts are used in various working conditions: at high and low temperatures and humidity, and with significantly high environmental levels of dust and salinity.

Technical requirements for bracket-type parts are specified in most literature sources, particularly in [25]. Traditionally, holes are used as the primary design datum and auxiliary design datum. They should be machined within the quality classes of IT6-IT11. The deviation of the center distances should not exceed 0.05−0.50 mm. The holes’ axes of the auxiliary design datum must be parallel or perpendicular to the holes’ axes of the main design datum, with permissible deviations from 0.02:100 to 0.10:100. The ends of the lugs of bracket-type parts and holes of the main design datum should be perpendicular to the axes of these holes in the range of 0.1:100 to 0.3:100, and the surface roughness of the ends should be Ra = 0.32–1.25 μm. Additionally, flat surfaces of the bracket-type parts must be perpendicular to the axes of the holes of the main design datum, with tolerances from 0.05:100 to 0.10:100, the roughness of their surfaces should be Ra = 0.63–2.50 μm. The roughness of the holes should be Ra = 0.63–2.50 μm, and the deviation from their shape should be within the tolerance for diameter (mainly 20–60%). The hardness of the material of fixture elements for bracket-type parts should be HRC 40–55, which increases their service life.

### 2.2. Design and Technological Classification

**Figure 1:**
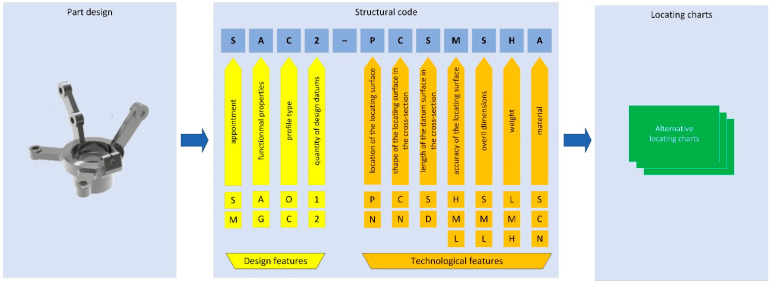
The transition from part configuration to the alternative locating charts.

The proposed design and technological classification [26] allow for the description of any bracket-type part by design and technological feature. As a result, four design and seven technological features were determined to be essential parameters for locating chart selections. This is an essential sub-step in transitioning from part configuration to generating alternative fixture layouts in a computer-aided design system. The general structure of the locating chart selection is presented in Figure 1.

According to the length of the locating surfaces, the parts are classified into brackets with long (*l*/*d* >1) and short (*l*/*d* <1) locating surfaces, which fundamentally determines the method of their locating during machining and, accordingly, the design of the fixture.

Bracket-type parts usually have one or more design data that are parallel or non-parallel to each other. Datum surfaces in cross-section can be round or non-round, which determines the shape of the locating surfaces of the locating elements.

By weight, the bracket-type parts made from steel (e.g., AISI 1A, 1010, 1045, and 3135; DIN GS-60) and cast iron (e.g., DIN 1691 and 1693-506-50) can be classified into light (less than 1 kg), medium (1–10 kg), and heavy (more than 10 kg), as well as for non-metallic materials, which affects the choice of equipment, cutting tools, and the appointment of cutting modes during machining.

Depending on the purpose, bracket-type parts are divided into parts with high (IT6–IT7), medium (IT8–IT10), and low (IT11–IT14) accuracy of locating surfaces. The machining of the locating surfaces with high accuracy, other things being equal, guarantees the more reliable and durable operation of the part and the product as a whole.

Considering the overall dimensions, bracket-type parts are divided into small (less than 50 × 50 mm), medium (from 50 × 50 to 300 × 300 mm), and large (more than 300 × 300 mm), which determines the overall dimensions of the fixture and the required working space of the machine tool during machining. The surface roughness of bracket-type parts is in the range of values Ra = 0.8–6.3.

Several theoretical locating charts can be proposed for each typical bracket-type representative. The choice of locating chart depends on the geometric shape and design features of the part (the presence of planes, ledges, holes, etc.), accuracy, size, shape, and spatial location of surfaces relative to each other, quality, roughness of surfaces, and stiffness (Table 1).

In practice, the number of implementation methods of locating charts can be extended according to the particular technical requirements and working conditions. After determining the most applicable implementation method, requirements for the set of locating elements are generated (e.g., dimension range of locating elements, type of work surface, etc.). The geometric shape, overall dimensions, and quality of locating surfaces are also considered when setting these requirements.

### 2.3. The Mathematical Model

The mathematical model for the rational choice of a locating chart is based on the following matrix equation:

(1) [Ψ]=[Ξ][W],

where [Ψ]—the matrix of locating charts; [Ξ]—the matrix of design and technological features, including materials; [*W*]—the matrix of transformation.

The matrix [Ψ] of locating charts is a rectangular one with the dimension of *N* × *m*, where *N*—the total number of considered parts; *m*—the total number of locating charts. The matrix [Ξ] of design and technological features is also a rectangular one, with the dimension of *N* × *n*, where *n*—the total number of design and technological features.

Notably, Equation (1) can be applied to the rectangular matrix [*W*] with the dimension of *n* × *m*. Its elements are determined based on previous practical experience in the rational design of *m* various locating charts for *N* typical parts. The corresponding expression for the matrix [*W*] can be written according to the following regression equation:

(2) $$\left[W\right]={\left({\left[\Xi \right]}^{T}\left[\Xi \right]\right)}^{-1}{\left[\Xi \right]}^{T}\left[\Psi \right],$$

Moreover, this expression is reliable for the case that the total number of considered parts exceeds the total number of selected locating charts (*N* ≥ *m*).

For the unification of the numerical calculation approach, it is advisable to use normalized values for all the elements of the matrices [Ψ]. This means that each element Ψ_*N*,*m*_ ranges from 0 to 1. For example, Ψ_*N*,*m*_ = 0 could mean that the *m*-th locating chart is not recommended for the *N*-th part. In this case, Ψ_*N*,*m*_ = 1 implies that the *m*-th locating chart is only recommended for the *N*-th part. Moreover, it is mandatory to follow the normalization rule:

(3) $$\underset{m}{\sum }\Psi_{N,m}=1$$

However, it is not mandatory to use this formula for the matrix [Ξ] due to the presence of several features within a single part. However, it is recommended that one use each element Ξ_*N*,*n*_ in a range from 0 to 1. If the *N*-th part has *k* ≥ 2 different features, the values of Ξ_*N*,*n*_ vary within the following variety $\left\{0,\frac{1}{k-1},\frac{2}{k-1},\ldots ,1\right\}$ of *k* rational numbers.

After evaluating the matrix [*W*], the normalized decision-making factor of the *m*-th locating chart for each *N*-th part is based on the column-vector {*Y*} of weighting factors determined as follows:

(4) $$Y_{m}^{\left(N\right)}=\frac{\left|{\left\{W^{\langle m\rangle }\right\}}^{T}\cdot {\left({\left\{\Xi \right\}}^{T}\right)}_{N}\right|}{\sum_{m}\left|{\left\{W^{\langle m\rangle }\right\}}^{T}\cdot {\left({\left\{\Xi \right\}}^{T}\right)}_{N}\right|}.$$

During decision-making for the *N*-th part, it is recommended that one choose the *j*-th locating chart with the maximum value of *Y*_*N*,*j*_ among all the values of *Y*_*N*,*m*_.

Finally, analogously to Formula (4), the decision-making for a part with *n* design and technological features different from any row of the matrix [Ξ] is determined as follows:

(5) $$\left\{Y\right\}=\frac{{\left[W\right]}^{T}\left\{X\right\}}{\sum_{m}{\left({\left[W\right]}^{T}\left\{X\right\}\right)}_{m}},$$

where {*X*}—the column-vector *n* × 1 of design and technological features of an arbitrary part and {*Y*}—the column vector *m* × 1 of the evaluated locating chart.

## 3. Results

**Figure 2:**
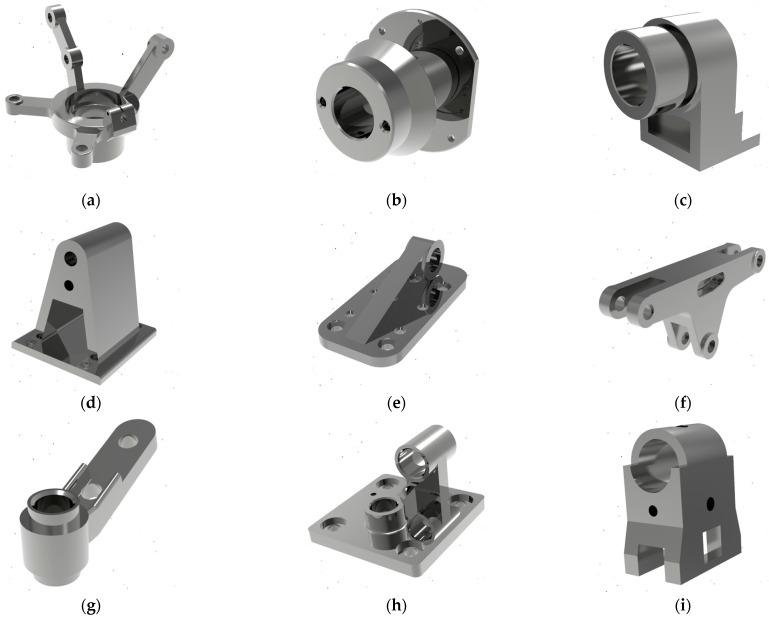

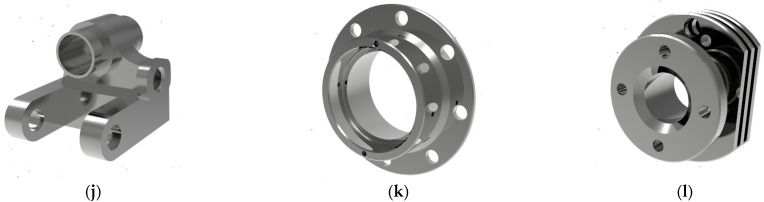
Typical representatives of bracket-type parts.

The analysis revealed the design of typical representatives of bracket-type parts (Figure 2). The defined parts differ in materials, geometric shape, number of surfaces, locating charts, and manufacturing technology.

**Figure 3:**
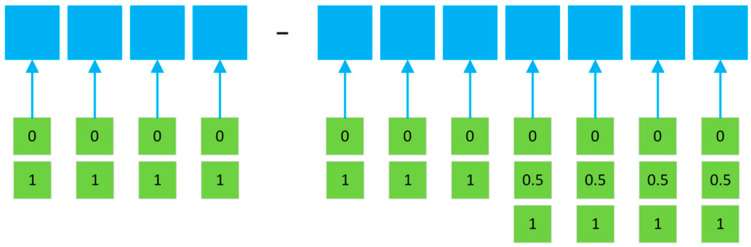
System of coding design and technological features for bracket-type parts.

The following parameters were used during the numerical calculations, according to the proposed mathematical model mentioned above. Notably, for evaluation of the matrix [*W*], a total number of parts *N* = 12 has been considered. The total number of design and technological features: *n* = 11 (Figure 3). For the first 7 of them, the number of features is *k* = 2; for the last *k*, *k* = 3.

The structural code was assigned to all selected bracket-type parts, considering the design and technological classifications. The locating chart was determined for all selected parts based on the surfaces that were to be machined. The developed model considers the different types of materials—parameter 11 in Figure 1 and Figure 3 (“S”—steel AISI 3135 and “C”—cast iron DIN 1691).

The above-mentioned information is summarized in Table 2.

During modeling, the various bracket-type parts composed of different materials (Table 2, column 2) were considered.

Therefore, the rectangular matrix {Ψ} of design and technological features (including materials) has a dimension of 12 × 11:

(6) $$\left[\Psi \right]=\left[\begin{array}{ccccccccccc} 0 & 1 & 0 & 1 & 1 & 0 & 1 & 0 & 0.5 & 0.5 & 0\\ 1 & 0 & 1 & 0 & 1 & 0 & 0 & 0.5 & 0.5 & 0.5 & 0\\ 0 & 0 & 1 & 1 & 0 & 0 & 1 & 0 & 0.5 & 0 & 0\\ 0 & 0 & 1 & 0 & 1 & 0 & 0 & 0 & 0.5 & 0.5 & 1\\ 0 & 0 & 1 & 0 & 1 & 0 & 1 & 0 & 1 & 0.5 & 0\\ 0 & 1 & 1 & 1 & 0 & 1 & 1 & 0 & 0.5 & 0.5 & 0\\ 1 & 0 & 1 & 0 & 1 & 0 & 1 & 0.5 & 1 & 0 & 0\\ 0 & 1 & 1 & 1 & 1 & 0 & 0 & 0 & 1 & 1 & 0\\ 0 & 0 & 1 & 0 & 0 & 0 & 1 & 0.5 & 0.5 & 0 & 0\\ 0 & 1 & 0 & 1 & 1 & 0 & 1 & 0 & 0.5 & 0.5 & 0\\ 0 & 1 & 0 & 1 & 0 & 0 & 0 & 0 & 0.5 & 0.5 & 0\\ 0 & 0 & 0 & 1 & 0 & 0 & 0 & 0 & 0.5 & 0.5 & 0 \end{array}\right]$$

Also, the total number of locating charts *m* = 6. Therefore, the rectangular matrix [Ξ] of locating charts has a dimension of 12 × 6

(7) $$\left[\Xi \right]=[\begin{array}{cccccc} 0 & 0 & 0 & 0 & 1 & 0\\ 0 & 1 & 0 & 0 & 0 & 0\\ 0 & 0 & 0 & 1 & 0 & 0\\ 0 & 0 & 0 & 0 & 0 & 1\\ 0 & 0 & 0 & 0 & 1 & 0\\ 0 & 0 & 0 & 1 & 0 & 0\\ 0 & 1 & 0 & 0 & 0 & 0\\ 0 & 0 & 0 & 1 & 0 & 0\\ 0 & 0 & 0 & 1 & 0 & 0\\ 0 & 0 & 1 & 0 & 1 & 0\\ 1 & 0 & 0 & 0 & 0 & 0\\ 0 & 1 & 0 & 0 & 0 & 0 \end{array}],$$

It should be noted that all of the columns have at least one non-zero value for the total consideration of all types of locating charts.

For each typical representative of a bracket-type part, calculations of the automatic selection of the rational locating chart according to the proposed approach, based on Equations (2) and (4), were performed. Particularly, the matrix of transformation is determined according to Equation (2):

(8) $$\left[W\right]=\left[\begin{array}{c} 0.571\;1.452\;0.351\;-1.774\;0.399\;-0.476\\ 0.571\;-0.714\;0.143\;0.143\;-0.143\;-0.143\\ 0.000\;-0.500\;-0.125\;0.75\;-0.125\;0.000\\ -0.143\;-0.071\;-0.161\;0.964\;-0.589\;0.286\\ -0.571\;-0.952\;-0.226\;1.024\;-0.274\;0.476\\ 0.714\;0.357\;-0.554\;1.321\;-0.696\;0.429\\ 0.143\;0.571\;0.536\;-1.214\;0.964\;-0.286\\ -0.286\;-0.143\;-0.321\;1.929\;-1.179\;0.571\\ 0.000\;0.000\;-0.500\;1.000\;-0.500\;0.00\\ 0.286\;2.143\;0.821\;-2.929\;1.679\;-0.571\\ 0.286\;0.473\;0.238\;-0.762\;-0.238\;0.762 \end{array}\right].$$

**Figure 4:**
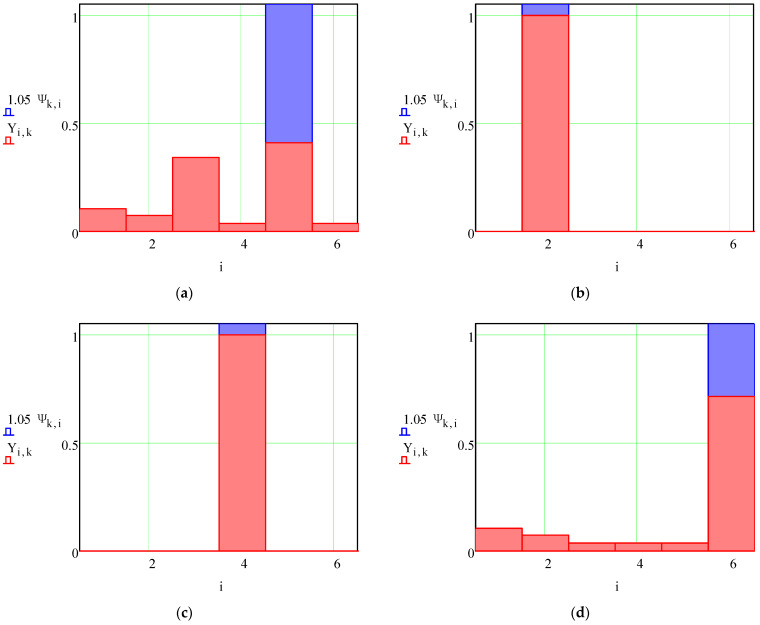

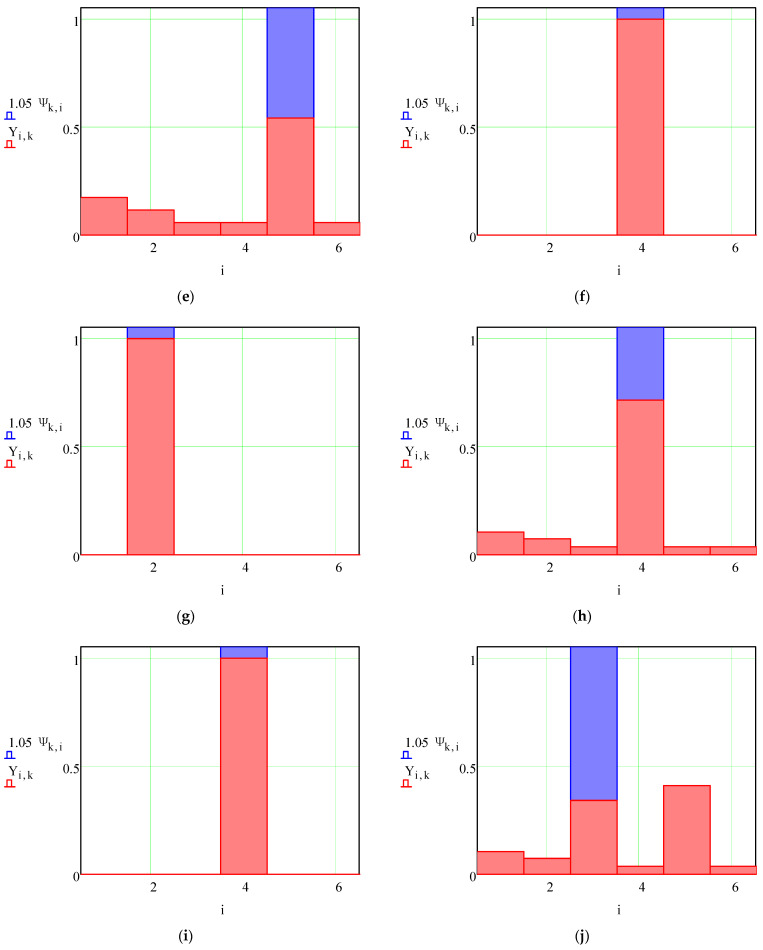

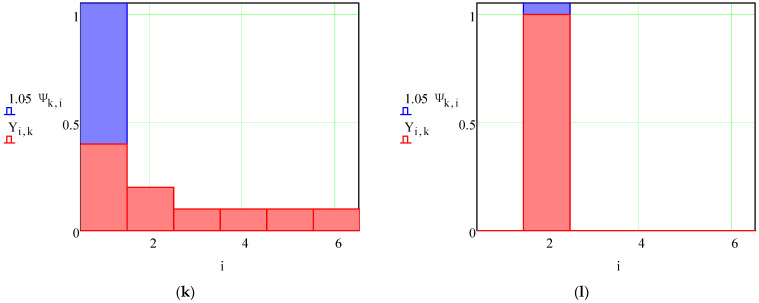
Calculations for locating chart selection: *i*—number of a locating chart (*i* = 1, 2, …, *m*); *Ψ_k_*_,*i*_—the recommended locating chart for *k*-th part (*k* = 1, 2, …, *N*); *Y_i_*_,*k*_—the evaluated weighing factor of *i*-th locating chart for the *k*-th part.

Also, the normalized decision-making factors 6 × 1 of locating charts for each part from the array of *N* = 12 parts was determined by Equation (4). Their values are summarized in Table 3 and graphically presented in Figure 4.

Analysis of the decision-making results is as follows. First, the numerical calculation results correspond to the assumed locating chart for all parts. This fact justifies the developed regression model (1)–(4) for decision-making in the rational choice of locating charts.

Second, for the 10th part, the decision-making approach proposes choosing the fifth locating chart despite the considered third one. Evidently, the locating chart using two planes and a hole is more appropriate for the part presented in Figure 2j. However, the final choice of the locating chart for this part depends on the surface under machining.

**Figure 5:**
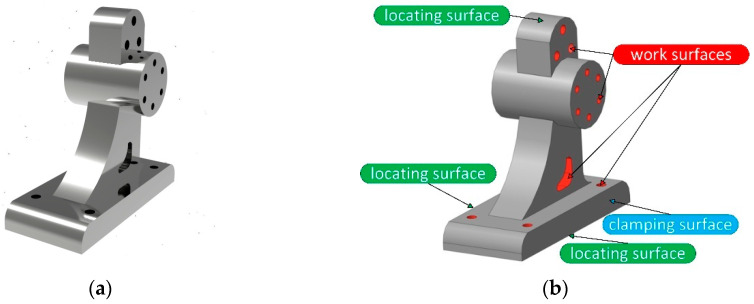

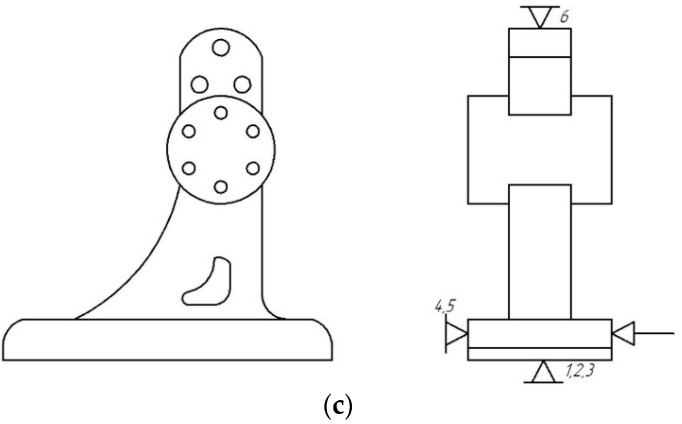
An arbitrary bracket-type part: (**a**), the functional surfaces (**b**), and the locating-and-clamping chart (**c**).

After evaluating the transformation matrix [*W*], the decision-making approach should be evaluated according to an arbitrary part. The design and technological features are different from previously considered data. As a particular case study, such a bracket-type part is presented in Figure 5a. In Figure 5b, the functional surfaces are presented. Particularly, the work surfaces are a combination of four different design and technological elements—three cylindrical holes, a cylindrical array of six holes located on the end, a groove, and four stepped holes on the down-plate of the part. Therefore, four different manufacturing steps should be applied in a single manufacturing operation. Thus, to realize the manufacturing operation, three surfaces will be used as locating surfaces, and one surface will be used as a clamping surface. From the practical point of view, the workpiece should be deprived of 6 degrees of freedom—2 perpendicular planes and an external cylindrical surface. To ensure the workpiece’s stable position during machining, the clamping force should be applied to the plane parallel to the datum (Figure 5c).

The structural code of the part (Figure 5) is “SAC1–NCSHLMA” (Figure 1 and Figure 3), and the column-vector of the design and technological features is as follows:

(9) $$\left\{X\right\}={\{\begin{array}{ccccccccccc} 0 & 0 & 1 & 0 & 1 & 0 & 0 & 0 & 1 & 0.5 & 0.5 \end{array}\}}^{T}.$$

Therefore, according to Formula (5), the evaluated column-vector {*Y*} of the locating chart is as follows:

(10) $$\left\{Y\right\}={\{\begin{array}{cccccc} 0.194 & 0.546 & 0.065 & 0.065 & 0.065 & 0.065 \end{array}\}}^{T}.$$

**Figure 6:**
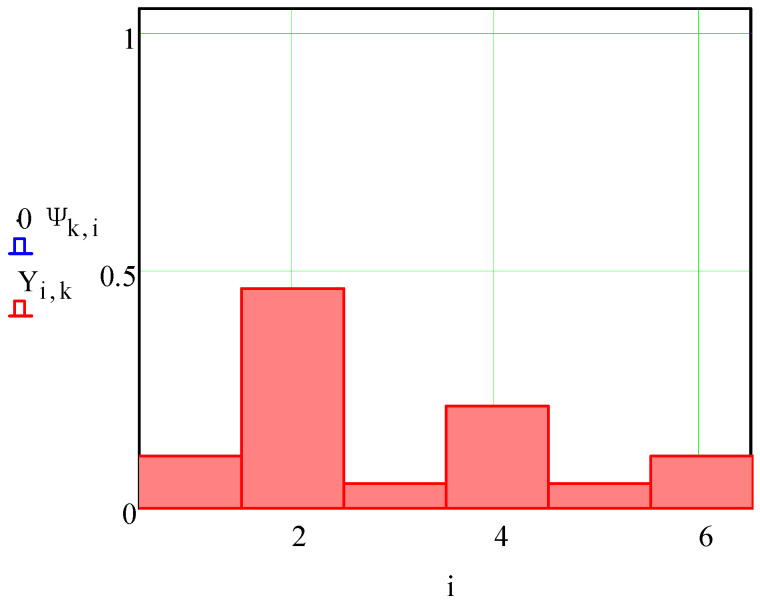
The normalized decision-making factors of the arbitrary part.

It is also graphically presented in Figure 6.

Since the second factor *Y*_2_ = 0.46 is the most valuable, the preferrable locating chart is No. 2—by an external cylindrical surface and two planes. The calculation results confirmed that, in practice, when machining bracket-type parts for similar machining conditions, the locating chart should be chosen by external cylindrical surface and plane.

## 4. Discussion

The scientific novelty of the present research is the development of a mathematical model for the rational choice of a locating chart by the design and technological features of the machined part based on the decision-making approach. Verification of the developed model was carried out on 12 examples of practical case studies and for an additional bracket-type part.

Analyzing the calculation results (Figure 4), three categories were distinguished. The first category applies when the calculated locating chart is the only one possible and coincides with the locating chart appointed by an expert (Figure 4b,c,f,g,i,l). The second category applies when the calculated locating chart is one of several possibilities, but the assigned expert assessment does not differ from the calculation results (Figure 4a,d,e,h,k). The third category of results corresponds to the production case study when, among several competitive locating charts, the assigned one differs from the calculations (Figure 4j). In this case, the final choice of the locating chart is made by a manufacturing engineer, considering the geometric and positional parameters of the work surfaces, machining parameters, and specified accuracy indicators.

The significance of the obtained results and their reliability is proven by their comparison with recent, globally produced results. Particularly, the locating system design module for freeform workpieces was developed for computer-aided fixture design [27]. Moreover, the mathematical model for optimal workpiece positioning in flexible fixtures was developed for thin-walled components [28]. The comprehensive mathematical justification of the locating process was presented in the research study [29].

As a result, a general scientific approach to the rational choice of locating chart for complexly shaped parts is based on the complex combination of design and technological features and a decision-making approach. The case study for bracket-type parts proves the reliability of this approach.

The practical significance of the obtained results is highlighted by the need to automate the fixture design process to reduce the time cost of the production planning stage. It is a crucial task in the implementation of the Industry 4.0 strategy.

Further research will be aimed at investigating the effect of locating errors.

## 5. Conclusions

The systematization of locating charts for workpieces in fixtures has been further developed. In particular, 6 locating charts and 23 implementation methods were identified for bracket-type parts manufacturing on CNC machining centers. Therefore, a scientific approach to the rational choice of the locating chart for complexly shaped parts has been developed to improve the fixture design process, including that performed in automated mode, based on a complex combination of the 11 design and technological features of the part and a decision-making approach.

The number of numerical simulations is not less than the total number of design and technological features during modeling. Moreover, the following limitations were used: the minimum weight of the part—1.2 kg, the maximum weight—2.9 kg; materials—steel and cast iron; an accuracy range of locating surfaces from H6 to H11; overall dimension ranges varied from 48 × 44 × 40 mm to 187 × 189 × 149 mm; the surface roughness was in the range of values: Ra = 1.25–3.2.

The developed methodology considers the different types of materials: steel AISI 3135 and cast iron DIN 1691. Its verification was completed using the example of bracket-type parts. Comparative analysis of the obtained results proved that the calculated solution corresponds to the variant assigned by an expert in the majority of cases. Practical implementation of the developed scientific approach was examined using bracket-type parts for the automotive industry.

Further development of the research is related to integrating the developed scientific approach into a computer-aided fixture design system, which will allow it to be integrated into intelligent manufacturing.

## Figures and Tables

**Table 1:** Possible implementation methods of theoretical locating charts.

Locating Chart		Implementation Method
1—by two external cylindrical surfaces and one plane	1.1	Self-centering V-blocks (3 DOF) + supports (3 DOF)
	1.2	V-blocks (4 DOF) + side support (1 DOF)
	1.3	V-block (2 DOF) + V-block (2 DOF) + side support (1 DOF)
2—by an external cylindrical surface and one or two plane(s)	2.1	Self-centering V-blocks (4 DOF) + side support (1 DOF)
	2.2	V-blocks (4 DOF) + side support (1 DOF)
	2.3	V-block (2 DOF) + V-block (2 DOF) + side support (1 DOF)
	2.4	Plate (3 DOF) + V-block (2 DOF) + side support (1 DOF)
3—by external cylindrical surface and a hole	3.1	Self-centering V-blocks (3 DOF) + supports (3 DOF)
	3.2	V-blocks (4 DOF) + support (1 DOF)
	3.3	V-block (2 DOF) + V-block (2 DOF) + support (1 DOF)
4—by three planes	4.1	Plates (3 DOF) + plate (2 DOF) + support (1 DOF)
	4.2	Supports (3 DOF) + supports (2 DOF) + support (1 DOF)
	4.3	Plates (3 DOF) + supports (2 DOF) + support (1 DOF)
	4.4	Plate & supports (3 DOF) + supports (2 DOF) + support (1 DOF)
	4.5	Plate & support (3 DOF) + plate (2 DOF) + support (1 DOF)
5—by two planes and one hole	5.1	Plates (3 DOF) + cylindrical pin (2 DOF) + support (1 DOF)
	5.2	Supports (3 DOF) + cylindrical pin (2 DOF) + support (1 DOF)
	5.3	Plates (3 DOF) + expanding pin (2 DOF) + support (1 DOF)
	5.4	Supports (3 DOF) + expanding pin (2 DOF) + support (1 DOF)
6—by one plane and two holes	6.1	Plates (3 DOF) + cylindrical pin (2 DOF) + cylindrical pin (1 DOF)
	6.2	Plates (3 DOF) + cylindrical pin (2 DOF) + diamond pin (1 DOF)
	6.3	Plates (3 DOF) + conical retractable pin (2 DOF) + diamond pin (1 DOF)
	6.4	Plates (3 DOF) + expanding self-centering pin (2 DOF) + diamond pin (1 DOF)

**Table 2:** Input data for calculations.

Part Design (Figure 2)	Part’s Structural Code (Figure 1 and Figure 3)	Locating Chart (Table 1)
a	SGO2–NCDHMMS *	5
b	MAC1–PCSMMMS	2
c	SAC2–PCDHMLS	4
d	SAC2–NCSHMHC *	6
e	SAC1–NCDHLMS	5
f	SGC2–PNDHMMS	4
g	MAO1–NCDMLLS	2
h	SGC2–NCSHLHS	4
i	SAC1–PCDMMLS	4
j	SGO2–NCDHMMS	3
k	SGO2–PCSHMMS	1
l	SAO2–PCSHMMS	2

* “S”—steel AISI 3135; “C”—cast iron DIN 1691.

**Table 3:** The normalized decision-making factors.

Part Design (Figure 2)	Number of the Decision-Making Factor					
	1	2	3	4	5	6
a	0.107	0.071	0.339	0.036	0.411	0.036
b	0.000	1.000	0.000	0.000	0.000	0.000
c	0.000	0.000	0.000	1.000	0.000	0.000
d	0.107	0.071	0.036	0.036	0.036	0.714
e	0.171	0.114	0.057	0.057	0.544	0.057
f	0.000	0.000	0.000	1.000	0.000	0.000
g	0.000	1.000	0.000	0.000	0.000	0.000
h	0.107	0.071	0.036	0.714	0.036	0.036
i	0.000	0.000	0.000	1.000	0.000	0.000
j	0.107	0.071	0.339	0.036	0.411	0.036
k	0.400	0.200	0.100	0.100	0.100	0.100
l	0.000	1.000	0.000	0.000	0.000	0.000

## Data Availability

The data presented in this study are available upon request from the corresponding author.
